# Dentition

**Published:** 1889-08

**Authors:** Walter A. Crow

**Affiliations:** Atlanta, Ga.; Lecturer on Physical Diagnosis and Diseases of Children. Southern Medical College


### DENTITION.

Portion of a lecture delivered in the Regular course of 1888—'89.

By WALTER A. CROW, B. S., M. D., ATLANTA, GA.

Lecturer on Physical Diagnosis and Diseases of Children. Southern Medical College.

GENTLEMEN:—To-day brings us to the consideration of *Dentition.* This is a subject of more than common interest to every physician, on account of the great number of cases with which he meets that are either directly or indirectly connected with the process of dentition. So severe may this disturbance become, that according to the mortality tables of London, as given by West, teething was assigned as the cause of death of 4.8% of all the children dying under one year old, and of 7.3% of those who die between the ages of twelve months and three years.

These are serious facts, and as I said is a subject of more than ordinary importance to you all. But whether dentition is wholly to blame for this vast number of troubles that complicate and follow in its wake is still a question among the ablest men of our profession. J. Lewis Smith says: "The opinion formerly entertained in the profession, and now prevalent in the community, that many infantile maladies arise directly or indirectly from dentition is erroneous." He claims that it is a physiological process and should be treated as such, while others are inclined to consider it pathological to some extent. In trying to decide upon this, there are a few facts that we should not lose sight of. We should remember that this includes the second great change in the life of the child—the first being at the time of birth, when it was separated from the mother and began to take air into its lungs and food into its stomach for its sustenance; and the next is the period of *dentition.* It is during this process as I have said, that the second great change in the child's life takes place, that is weaning. The child will soon be deprived of the mother's breast for its nourishment, and will begin to take solid food, which it will have to masticate and partly digest, before it goes into the stomach. It is a period of active organic processes; changes are going on throughout the whole system, including the nervous, vascular, respiratory and alimentary systems. Hence there must be an unusual susceptibility to morbid influences.

I am confident I have seen a number of pulmonary and gastro-enteric troubles that were greatly aggravated by the appearance of a swollen gum, while on the other hand I am equally as confident that I have seen convulsive attacks and other grave nervous troubles, that were due to nerve irritations from the inflamed gum, stop when the little tooth was fairly through. So I am inclined to consider the process of dentition a physiological one, and should be treated as such, but on account of the peculiar conditions of the organism I can see how easy it is for a pathological condition to take place.

The first teeth, which are usually the lower incisors, generally make their appearance about the sixth month: in some it may be as early as the fourth month, while in others as late as the tenth or eleventh month. Next after the lower incisors, usually from four to six weeks, the upper incisors come through, then following in succession, we have the upper and lower lateral incisors, the four anterior molars, the four canines, and lastly, the four posterior molars, but they do not always follow in this order.

The incisors usually appear in rapid succession, and are all in sight by the end of the first year. From the age of one year to sixteen months the anterior molars appear, and from sixteen months to two years the canines, and by the end of the next six months, thirty months, the posterior molars are usually through—this completes the first dentition or milk teeth.

There are a few rare cases in which the child has had teeth when born, such a case I believe, was reported to the Virginia Society of Dentists a year or so ago. Retarded dentition is by no means uncommon, especially so with children that suffer from rickets, or are feeble from constitutional taint, or the result of disease.

It is in this class that we are more especially apt to have some constitutional disturbances; and now whether these are coincidences, or due to mechanical causes, or of reflex nervous origin, due to the combined sympathy between the great vagus, the sympathetic system and and the extremely sensitive fifth nerve, are questions of conjecture that suggest themselves to the mind of every earnest thinker.

We cannot ignore the nervous factor in a great many of these cases, especially when we consider the long list of troubles that are apt to be connected with this process, such as catarrhal otalgia, conjunctivitis, stomatitis, gastritis, gastro-enteritis, enteritis, entero-colitis, cholera-infantum, from a slight cough to a severe bronchitis or pneumonia, the smile that plays on the face of the sleeping child, due to contraction of the facial muscles, to the severest type of tonic or clonic convulsions, or the array of cutaneous eruptions, such as: Uticaria, eczema, impetigo, lichens, prurigo, herpes, &c. These are often perplexing complications, gentlemen, that may give you no little trouble when you meet with them in your treatment of the little ones.

The first evidence of dentition in the child is usually manifested by an excess of saliva; these glands that are inactive during the first few months now begin to secrete an excess of fluid that often runs out the corners of the mouth over the chin, so as to wet a good portion of the dress in front. The gums also begin to flatten and get broader, the child is usually more or less fretful, but not necessarily so, for sometimes the mother to her delight, finds the little tooth already through. But in the majority of cases as I said, you will find the child more or less fretful, it does not sleep so well as usual, bowels perhaps a little looser, it will have hot flashes, accompanied with more or less fever, and most always there is a constant desire to press the swollen gum on something, for they usually keep their finger in the mouth, or anything they can get hold of. This pressing against the gums seems to give temporary relief, and for this reason it is well enough to supply the child with a rubber or ivory ring. I do not approve of the practice of a great many mothers, of rubbing the swollen, tender gum of the child with a thimble, or some hard substance, in order to hasten the appearance of the teeth, for this only adds irritation to the already highly inflamed gum.

Treatment:—This is carried on, on general principles, depending entirely on the nature of the complication. If there should be any disorder of the stomach or bowels, it is always a good plan to clear them of any irritating substance that may be lodged therein, and for this purpose I know nothing better than the "old time" medicine of our mothers and grandmothers, that is, castor oil and turpentine, or rhubarb and soda bicarb. After you have succeeded in removing all the offending substances, then pursue your treatment P. R. N. If any irritation should still remain, try to quiet it by soothing applications or medications; if the digestive organs are out of order, try and correct them. If there should be a tendency to acidity with more or less irritability, and too much looseness of the bowels, I usually prescribe sub. nit. bismuth, gr. v., to ʒi milk magnesia (Phillip) with or without tr. opii camph., as the case may be. To this same mixture I have sometimes, in cases of weak, delicate children, added syr. lactophosphate lime with a decided advantage. But gentlemen, so much of your success in the treatment of these cases depends upon your ability to recognize and meet the indications promptly, that I shall not attempt to give you any specific rules for treatment. In those cases where there is gastric or intestinal trouble, you will likely do a great deal of good in the way of prophylaxis, by protecting the stomach and bowels from atmospheric changes, for this reason I usually insist on the wearing of a broad flannel binder over the stomach and abdomen.

The food if artificial should be modified to some extent, it is often best to temporarily reduce the quantity, and render it as easily digested as possible. Farinaceous articles should be restricted or entirely forbidden in some cases, on account of the tendency to acid fermentation.

It is a prevalent idea among a great many that some looseness of the bowels is rather favorable than otherwise, this I think depends entirely upon the character of the discharges. If they present a healthy appearance without any mucous, and not accompanied with pain, and not more than three evacuations in twenty-four hours, then I rarely interfere, for I think it is nature's effort to relieve the nervous tension, and I consider the condition somewhat similar to that which we often see in adults, which is brought on by excitement or prolonged nervous strain, &c. There is also another point to which I wish to refer before closing, that is the practice of lancing the gums, which has been considered as a favorite remedy for almost all disorders at this period. While I do not condemn the practice altogether, still I think that there are specific indications. They are the tight, swollen gums on which the tooth is firmly pressing, so that when the gum is divided it will retract, thus protruding the point of the tooth. If you should lance the gums before there is this tension on them, the incision will likely heal up, and you will make bad matters worse by having the tooth to cut through a circatrix instead of the normal gums. But by judicious lancing you can often give great relief. I have often been called to see a child that was crying and fretting all the time. Its sufferings seemed so great that it could neither eat nor sleep, and by relieving the tension of the highly inflamed gum, by cutting down on the little tooth, it would stop crying at once, and in fifteen or twenty minutes would be sound asleep, and perhaps would have no return of the trouble whatever.

### SULPHUR FUMIGATION.

A writer in *Brooklyn Medical Journal* remarks: The medical faculty can scarcely blame ordinary mortals for holding their minds in a solution of doubt as to whether there are any disinfectants known to science which really disinfect. The few opportunities I have had of observing the experiments in disinfectants may perhaps have been no fit test. They may not have been sufficiently thorough in their application, as in a case I recollect which occurred in the city of Perth in Scotland, when some cases of cholera caused considerable alarm among the people. The provosts and magistrates held a meeting, the wise men of the city took counsel together, and lo! two alleyways were straightway whitewashed. The cholera gradually vanished, but it was in no hurry about it.

A recent experience in my own house might not be uninteresting to your readers. Last October one of our children was taken with scarlet fever. The doctor advised us to send two of the younger children away, and by confining the sick child to a room in the third story of the house lessen the chances of infection. The instructions were immediately obeyed. The two children were absent from the house nearly three months. The sick child had entirely recovered, and a system of fumigating was ordered. Everything was removed from the room where the sick child had been; in fact, from two rooms, the parlor and bedroom immediately adjoining. Carpets and other material were burned. A quantity of dry sulphur was burned in the rooms, about two pounds, which kept the rooms in a state of suffocation for several hours. Then a complete washing of floors and every article of furniture carefully performed, the walls and ceilings being thoroughly cleaned, nearly a gallon of carbolic acid being used in the operation. The bedstead and other movables were revarnished, and after a readjustment of material another burning of sulphur, about 1 pound, was proceeded with. A considerable quantity of sulphur was also used in the kitchen on the ground floor, and every suggestion of our medical adviser was punctually attended to.

The sulphur was burned partly in a coal-shovel set in the stove, the door of the stove being open and the damper in the stovepipe shut, and another quantity in the other room in a smaller shovel set in a coal-scuttle. No water was used, and there was no water in the rooms at the time; this I am sure of. The burning of the sulphur was exactly as the doctor had instructed us. The area of the floor, 36x18 =648, about 11 feet high, = 7128 cubic feet, minus the hallway, 6x 24x11=1584=5544, or about 5,000 feet divided into three rooms. I am not quite positive that the quantity burned on the first occasion amounted to two pounds. Possibly one-half pound was used in the kitchen, which would make one and one-half pounds in the two rooms, or probably more. On the second occasion the quantity used was perhaps not more than half as much. We were not particular about the quantity. There was more used than the doctor thought sufficient.

The doctor recommended leaving the windows open during some of the coldest days in December, and two days particularly the temperature in the rooms was at least ten degrees below the freezing-point. After the doctor had assured us that it was perfectly safe to bring the children home, we burned another quantity of sulphur, about one-half pound, dry, and delayed the coming home about a week.

They came home on Christmas Day, and, as you may imagine, it was a kind of merry Christmas with us. Just eight days after, one took the fever, and on the third day succeeding the other followed. One peculiarity was that neither of the latter cases resembled the first; for while the first case had the fever accompanied with intermittent delirium, the other two were quite rational but had severe affections of the throat. One of the latter cases was particularly serious, the doctor hardly expecting recovery. They all recovered.

I may add that during the period the latter two were afflicted, every one of the house was more or less affected with sore throat, even two or three visitors also becoming similarly affected.

I cannot, therefore, place much faith in dry sulphur or carbolic acid as disinfectants. It would have been a great saving of trouble to us if the children had been permitted to stay at home and had the disease together. We did the best we could. Since their complete recovery we have not bothered about any more sulphur.

